# 

**Published:** 1891-05

**Authors:** 


TO CONTRIBUTORS.

Articles for publication must reach this office not later than 15th inst.; and are expected to be published exclusively in this JOURNAL. Extra copies of JOURNAL furnished contributors when requested. Reprints by special arrangement. Publication of an article does not necessarily imply endorsement of the views therein expressed.

MEDICAL AND SURGICAL JOURNAL, Post-office box 431.

### MEETING OF THE NATIONAL ASSOCIATION OF RAILWAY SURGEONS.

At the Kansas City meeting of the National Association of Railway Surgeons last year, it was decided to hold the next meeting at Buffalo, N. Y., May 7th, 8th and 9th of this year. But, on account of the meeting of the American Medical Association being set for the same time, it has been decided to change those dates, and to hold our next meeting at Buffalo April 30th and May 1st and 2d, to which all railway surgeons are cordially invited. To all railway surgeons sending their names and addresses to the corresponding secretary, a copy of the constitution and programme will be sent. All those wishing to read papers should send in the titles of their papers without delay. For further information inquire of

A. G. GUNMAER,

Corresponding Secretary,

Buffalo, N. Y.

CONGRESS OF AMERICAN PHYSICIANS AND SURGEONS.—The meeting of the Congress of American Physicians and Surgeons will be held in Washington from 3 to 6 P. M., September 22d, 23d, 24th and 25th, 1891. William Pepper, Chairman of the Executive Committee.

